# Medical Record System Redesign to Enhance Efficiency and Safety in Psychiatric Inpatient Care

**DOI:** 10.1192/bjo.2025.10380

**Published:** 2025-06-20

**Authors:** Abeer Jawed, Anjani Atigadda, Rahimat Adebayo, Lara Jayatilaka, Solomon Wong

**Affiliations:** Central and North Western London NHS Foundation Trust, London, United Kingdom

## Abstract

**Aims:** Efficient medical record systems are vital for improving workflow, ensuring data accuracy, and maintaining staff satisfaction. At Eastlake and Ferneley psychiatric wards, the medical record system, ‘SystmOne’ proved to be inefficient and fragmented, with essential functions scattered across interfaces. This led to excessive administrative burden, detracted from patient care, and frustrated staff. A pre-implementation survey revealed issues such as excessive time spent on documentation and difficulties locating multidisciplinary input, underscoring the need for systemic change. The aim of this project was to reduce administrative time and improve user satisfaction by 30% by December 2024 through a consolidated visualisation tool.

**Methods:** A before and after study was carried out and Plan-Do-Study-Act (PDSA) cycle methodology was used. A new consolidated visualisation tool was designed to streamline key workflows, including admissions, ward activities, and discharges. Healthcare professionals (doctors and nurses) working across two psychiatric wards were participants on this study. Baseline data was collected including quantitative data on time taken to do daily tasks, and number of clicks; as well as qualitative data in the form of user satisfaction surveys. Training sessions were carried out to enable the staff to proficiently use the visualisation, followed by a roll-out of the visualisation on SystmOne. Post-implementation metrics were collected, including the number of clicks, time per task, and user feedback from follow-up surveys to evaluate the intervention’s impact.

**Results:** The intervention resulted in a 92% reduction in clicks and a 62% reduction in time taken to do daily tasks for doctors, and an 89% reduction in clicks and 87% reduction in time for nurses. Pre-implementation, most tasks took 35–40 minutes, whereas post-implementation tasks were completed in under 5 minutes. Annually, this equated to saving 304 hours for doctors and 440 hours for nurses. Qualitative feedback emphasized ease of use, reduced errors through a traffic-light system, and improved data accessibility. Staff reported increased job satisfaction and less frustration, allowing more time for patient care.

**Conclusion:** The system redesign significantly enhanced workflow efficiency, data quality, and user satisfaction, with clear implications for improved patient safety and care within psychiatric inpatient wards. These findings highlight the value of streamlining documentation systems to reduce administrative burden, enhance staff well-being, and foster patient-centred care. Wider rollout of the tool is recommended, alongside iterative refinements and ongoing evaluations to ensure sustainability and adaptability to evolving clinical needs.

